# Reproducible research practices, transparency, and open access data in the biomedical literature, 2015–2017

**DOI:** 10.1371/journal.pbio.2006930

**Published:** 2018-11-20

**Authors:** Joshua D. Wallach, Kevin W. Boyack, John P. A. Ioannidis

**Affiliations:** 1 Department of Environmental Health Sciences, Yale School of Public Health, New Haven, Connecticut, United States of America; 2 Collaboration for Research Integrity and Transparency, Yale School of Medicine, Yale University, New Haven, Connecticut, United States of America; 3 SciTech Strategies, Inc., Albuquerque, New Mexico, United States of America; 4 Stanford Prevention Research Center, Department of Medicine, Stanford University, Stanford, California, United States of America; 5 Department of Health Research and Policy, Stanford University, Stanford, California, United States of America; 6 Department of Biomedical Data Science, Stanford University, Stanford, California, United States of America; 7 Department of Statistics, Stanford University, Stanford, California, United States of America; 8 Meta-Research Innovation Center at Stanford, Stanford University, Stanford, California, United States of America; Charité—Universitätsmedizin Berlin, Germany

## Abstract

Currently, there is a growing interest in ensuring the transparency and reproducibility of the published scientific literature. According to a previous evaluation of 441 biomedical journals articles published in 2000–2014, the biomedical literature largely lacked transparency in important dimensions. Here, we surveyed a random sample of 149 biomedical articles published between 2015 and 2017 and determined the proportion reporting sources of public and/or private funding and conflicts of interests, sharing protocols and raw data, and undergoing rigorous independent replication and reproducibility checks. We also investigated what can be learned about reproducibility and transparency indicators from open access data provided on PubMed. The majority of the 149 studies disclosed some information regarding funding (103, 69.1% [95% confidence interval, 61.0% to 76.3%]) or conflicts of interest (97, 65.1% [56.8% to 72.6%]). Among the 104 articles with empirical data in which protocols or data sharing would be pertinent, 19 (18.3% [11.6% to 27.3%]) discussed publicly available data; only one (1.0% [0.1% to 6.0%]) included a link to a full study protocol. Among the 97 articles in which replication in studies with different data would be pertinent, there were five replication efforts (5.2% [1.9% to 12.2%]). Although clinical trial identification numbers and funding details were often provided on PubMed, only two of the articles without a full text article in PubMed Central that discussed publicly available data at the full text level also contained information related to data sharing on PubMed; none had a conflicts of interest statement on PubMed. Our evaluation suggests that although there have been improvements over the last few years in certain key indicators of reproducibility and transparency, opportunities exist to improve reproducible research practices across the biomedical literature and to make features related to reproducibility more readily visible in PubMed.

## Author summary

Currently, there is a growing interest in ensuring the transparency and reproducibility of the published scientific literature. According to a previous evaluation of 441 biomedical articles published from 2000–2014, the majority of studies did not share protocols and raw data or disclose funding or potential conflicts of interest. However, multiple recent efforts, which are attempting to address some of the existing concerns, may be resulting in genuine improvements in the transparency, openness, and reproducibility of the scientific literature. In this study, we investigate the reproducibility and transparency practices across the published biomedical literature from 2015–2017. We analyze reporting of public and/or private funding and conflicts of interests, sharing protocols and raw data, and independent replication and reproducibility checks. We also investigate what can be learned about reproducibility and transparency indicators from open access data provided on PubMed. Our evaluation suggests that although there have been improvements over the last few years in some aspects of reproducibility and transparency (e.g., data sharing), opportunities exist to improve reproducible research practices across the biomedical literature and to make features related to reproducibility more readily visible in PubMed.

## Introduction

There is a growing interest in evaluating and ensuring the transparency and reproducibility of the published scientific literature. According to an internet-based survey of 1,576 researchers in *Nature*, 90% of respondents believe that there is either a slight or significant crisis of reproducibility in research [1]. However, multiple recent efforts are attempting to address some of the existing concerns [2–6]. These initiatives, as well as previous proposals by several stakeholders to change scientific practice, may be resulting in genuine improvements in the transparency, openness, and reproducibility of the scientific literature.

A survey of a random sample of biomedical articles published from 2000–2014 suggested that the literature lacked transparency in important dimensions and that reproducibility was not valued appropriately [7]. For instance, protocols and raw data were not directly available, and the majority of studies did not disclose funding or potential conflicts of interest. Furthermore, over half of the articles in the sample claimed to present some novel discoveries and the vast majority did not have subsequent studies that were attempting to replicate part or all of their findings [7]. These results suggested that there is significant room for improvement with regard to reproducible research practices. Furthermore, the study provided baseline data to compare future progress across key indicators of reproducibility and transparency.

Since 2014, there have been new or intensified efforts to promote open science practices across the biomedical literature. Although it is unlikely that individual interventions have single-handedly resulted in drastic changes, these efforts may cumulatively reflect a gradual shift toward the adoption of a culture that embraces transparency and replication. For instance, in January 2015, the Institute of Medicine issued a report that recommended that all stakeholders in clinical trials “foster a culture in which data sharing is the expected norm,” and that funders, sponsors, and journals promote and support data sharing [8]. The International Committee of Medical Journal Editors (ICMJE) also proposed a policy requiring data sharing as a condition of publication, even though no formal policy changes have been enacted [9, 10]. Other stakeholders have also supported raw data sharing [2] and some journals have started requesting full protocol sharing [11], since access to detailed protocols is necessary to allow study procedures to be repeated [12]. Several fields are paying more attention to replication, especially after the findings of reproducibility checks demonstrated concerning results in psychology [13] and cancer biology [14, 15]. Furthermore, a growing number of journals have started to require reporting guidelines and disclosure statements, and commercial and nonprofit organizations, such as the Open Science Framework (http://osf.io), have introduced new infrastructure supporting research transparency.

Additional efforts have also tried to improve the disclosure and visible indexing of information related to transparency and reproducibility. In 2017, PubMed, which is run by the United States National Library of Medicine (NLM) at the National Institutes of Health (NIH), started including funding and conflicts of interest statements with study abstracts. Although this information is often disclosed in the full text of journal articles, many research consumers do not have a subscription to all of the journals catalogued in PubMed. To our knowledge, it is unknown whether information about key transparency indicators is easily accessible to the general public on PubMed and whether this information was available prior to 2017.

These and other recent open science initiatives, or even simply the wider sensitization of the scientific community over the past 20 years, may have improved the reproducibility and transparency of the biomedical research over the last few years. However, to our knowledge, there is no evidence on whether progress has been made on all, some, or none of the indicators that have been proposed as being important to monitor [5, 7].

Given the importance of examining the progress of reproducibility and transparency in the scientific literature, we sought to build upon our previous analysis [7] and to assess the status of reproducibility and transparency in a random sample of biomedical journal articles published between 2015 and 2017. Here, we evaluate the proportion of studies reporting sources of public and/or private funding and conflicts of interest, sharing protocols and raw data, and undergoing rigorous independent replication and reproducibility checks. We also investigate what can be learned about these reproducibility and transparency indicators from widely accessible open access data provided on PubMed.

## Results

### Description of assessed sample of articles, 2015–2017

Among the 155 randomly selected articles published between 2015 and 2017, we excluded 6 non-English language articles. Of the remaining 149, 68 (45.6% [95% confidence interval, 37.5% to 54.0%]) were publications in the research field of Medicine, with smaller numbers in the fields of Health Sciences (*n* = 28), Biology (*n* = 13), Infectious Disease (*n* = 16), and Brain Sciences (*n* = 24). Among 120 articles that were published in a journal with a 2013 impact factor, the median impact factor was 3.1 (interquartile range, 2.0–4.7).

The majority of publications had some form of empirical data (118 of 149 [79.2% (95% confidence interval, 71.6% to 85.2%)]—*n* = 104 excluding case studies and case series, in which protocol and raw data sharing may not be pertinent, and *n* = 97 excluding also systematic reviews, meta-analyses and cost-effectiveness analyses in which replication in studies with different data would not be pertinent). Among the 149 eligible articles, there was one (0.7% [0.0% to 4.2%]) cost-effectiveness or decision analysis, 14 (9.4% [5.4% to 15.6%]) case studies or case series, four (2.7% [0.9% to 7.2%]) randomized clinical trials, six (4.0% [1.6% to 8.9%]) systematic reviews and/or meta-analyses, and 92 (61.7% [53.4% to 69.5%]) “other” articles with empirical data (including cross-sectional, case-control, cohort, and various other uncontrolled human or animal studies). Approximately one-fifth (20.8% [14.8% to 28.4%]) of the sample was classified as research without empirical data or models/modeling studies. There were 64 (43.0% [35.0% to 51.3%]) with a PubMed Central reference number (PMCID), of which 37 were also PubMed Central Open Access (PMCOA).

### Funding

Nearly one-third (46, 30.9% [23.7% to 39.0%]) of the 149 biomedical articles did not include information on funding. There were 78 articles (52.3% [44.0% to 60.5%]) that were publicly funded, either alone or in combination with other funding sources. Of these, three received National Science Foundation (NSF) support and 25 had NIH funding, either alone or in combination with other funding sources.

### Reporting of conflicts of interest

Among the 149 articles, there were 52 (34.9% [27.4% to 43.2%]) that did not include a conflicts of interest statement. However, there were 87 (58.4% [50.0% to 66.3%]) that specifically reported no conflicts of interests and 10 (6.7% [3.4% to 12.3%]) that included a clear statement of conflict.

### Protocol and raw data availability

Excluding case studies or case series and models/modeling studies, in which a protocol would not be relevant, one (1.0% [0.1% to 6.0%]) of the 104 articles with empirical data included a link to a full study protocol. This article was a systematic review that stated that “methods for study inclusion and data analysis were prespecified in a registered protocol (PROSPERO 2015:CRD42015025382)” (PMID: 27863164) [16]. There was also one clinical trial (27391533) and two prospective cohort studies (25682436 and 28726115) that referenced a ClinicalTrials.gov identifier (i.e., an NCT number). For two of the studies (27391533 and 28726115), the month and year in which sponsors or investigators first submitted a study record to ClinicalTrials.gov were the same as the reported study start dates. For one of the observational studies (25682436), the first ClinicalTrials.gov study record date was approximately 11 years after the disclosed study start date.

There were 31 (29.8% [21.4% to 39.7%]) articles that included supplemental materials, including methods sections, videos, tables, survey materials, and/or figures, either as a detailed appendix at the end of the article or online. However, none of the supplementary materials allowed for a reconstruction of a full protocol. Furthermore, none of the articles mentioned any sharing of scripts/code.

There were 19 (19 of 104, 18.3% [11.6% to 27.3%]) articles that discussed some level of publicly available data (Table 1**)**. While 13 provided data set identifiers or accession codes, there were four articles that included supplementary excel data files. Although another article mentioned that all relevant data were within the supporting information files, the supplementary files did not contain any raw data (26413900).

**Table 1:** Data sharing characteristics among 19 biomedical articles with a data sharing statement.

PMID*	Data statement	Category^a^	PubMed^b^	Functioning^c^
26484203*	“Gene Expression Omnibus (GEO) database repository with the dataset identifier GSE63072.”	Identifiers/accession numbers	Yes	Yes
27096608*	“All data are made available on a public repository (OpenfMRI, accession number ds000202). All other relevant data are added to the text as supplementary material.”	Identifiers/accession numbers	No	Yes
27348411*	“NCBI Sequence Read Archive: TCR sequence data, PRJNA324707 and PRJNA325247. Supplementary excel files with additional data.”	Identifiers/accession numbers; excel data	Yes	Yes
27617276*	“Raw data derived from this analysis have been deposited to the ProteomeXchange Consortium via the PRIDE partner repository with the dataset identifier PRIDE: PXD002768.”	Identifiers/accession numbers	Yes	Yes
28970499*	“The dataset generated during and/or analysed during the current study are available from the corresponding author on reasonable request.”	Upon request	No	N/A
28632753*	“All relevant data are within the paper and its Supporting Information files.”	Excel data	No	Yes
28241009*	“All relevant data are within the paper and its Supporting Information files. The accession codes for LSSmCherry1 and RDSmCherry1 are KX638424 and KX638425, which can be viewed here: https://www.ncbi.nlm.nih.gov/genbank/.”	Identifiers/accession numbers; excel data	No	Yes
28886694*	“Sequence data of 15 RNA-seq have been uploaded to the NCBI database, and the SRA number was SRX2843778.”	Identifiers/accession numbers	No	No
27214551	“Supplemental material available online with this article.”	Primers used for qPCR analyses; excel data	No	Yes
27791002	“The data reported in this paper have been deposited in the Gene Expression Omnibus (GEO) database, www.ncbi.nlm.nih.gov/geo (accession no. GSE86536).”	Identifiers/accession numbers	No	Yes
26238763	“The mass spectrometry proteomics data have been deposited to the ProteomeXchange Consortium via the PRIDE partner repository with the dataset identifier PXD001593 and 10.6019/PXD001592.”	Identifiers/accession numbers	No	Yes
27768894	“The accession number for the coordinates and structures factors of CB1_AM6538 reported in this paper is PDB: 5TGZ.”	Identifiers/accession numbers	No	Yes
25252277	“We demonstrate SCoTMI on publicly available resting-state fMRI data from the Human Connectome Project.”	Public data	No	No
26639818	“*ITBG2* sequence variants identified in this study have already been submitted to GenBank allocated with 31 accession numbers from KJ528562 to KJ528592…Full data are also accessible using URL mentioned below; http://www.ncbi.nlm.nih.gov/nuccore?term=yassaee.”	Identifiers/accession numbers	No	Yes
27871817	“Collection data and GenBank accession numbers for *Proctoeces* taxa sequenced for this study are presented in Table 2…Newly generated 18S and 28S rDNA sequences were aligned with sequences of species of *Proctoeces* and other fellodistomid taxa available on GenBank (Table 2).”	Identifiers/accession numbers	Yes	N/A
28349993	“The genotype data of the 1000 Genomes Project Phase 1 based on 1,092 healthy subjects—525 male (48.1%) and 567 female (51.9%; www.1000genomes.org) were used as the control group.”	Data link	No	Yes
28528644	“Gene expression array data will be provided or personal research purposes through the corresponding author; residual tissues from the studies may be applied for through the Tayside Tissue Bank, Dundee, Scotland.”	Upon request	No	N/A
28412520	“The accession number for the RNA-sequencing and whole-genome sequencing data reported in this paper is Sequence Read Archive: SRP100435.”	Identifiers/accession numbers	No	Yes
27108998	“The complete genome sequence of SAIBK2 obtained in this study was submitted to Genbank database under the accession number of KU317090.”	Identifiers/accession numbers	No	Yes

* PubMed Central Open Access (PMCOA) articles.

^a^ Category of data sharing.

^b^ Data sharing information available at the abstract/PubMed level.

^c^ Were the links, identifiers, or accession numbers functioning?

**Abbreviations:** N/A, not applicable; PMID, PubMed identification number.

### Articles claiming to contain novel findings versus replication efforts

Among the 97 biomedical articles with empirical data, excluding case studies and case series, systematic reviews/meta-analyses, and cost effectiveness/decision analyses studies, only five (5.2% [1.9% to 12.2%]) were inferred to be replication efforts trying to validate previous knowledge. Over half (56, 57.7% [47.3% to 67.6%]) claimed to present some novel findings. Although 10 (10.3% [5.3% to 18.6%]) articles had statements of both study novelty and some form of replication, 26 (26.8% [18.6% to 36.9%]) had no statement or an unclear statement in the abstract and/or introduction about whether the article presented novel findings or replication efforts.

### Subsequent citing by replication studies

Of the 97 biomedical articles with empirical data, there were two articles that had at least some portion of their findings replicated. One of the replicating articles used an “almost comparable study design but over a longer period” and included some patients with different characteristics (Index article: 24415438, replication: 27363404) [17]. The second was a partial replication effort with a longer follow-up (Index article: 27067885, replication: 27241577). Only one article was included in a subsequent systematic review.

### Comparison based on PMCID and PMCOA status

As shown in Table 2, there were no statistically significant differences between PMCOA and non-PMCOA articles or between articles with a PMCID or without a PMCID. However, there was a suggestion for fewer articles having a statement of no conflicts of interest (*p* = 0.014) and more articles including a statement pertaining to data sharing (*p* = 0.049) in the PMCOA group than in the non-PMCOA group. Furthermore, there was a suggestion that articles without PMCIDs were less likely to mention funding and to have public funding than in the PMCID group (*p* = 0.015).

**Table 2:** Articles in the PMCOA versus non-PMCOA and PMCID versus non-PMCID categories.

Variable^a^	PMCOA		Non-PMCOA		*P* ^b^	PMCID		Non-PMCID		*P*^b^
	*N*	%	*N*	%		*N*	%	*N*	%	
**Funding**	***N* = 37**		***N* = 112**			***N* = 64**		***N* = 85**		*
No Mention	10	27.0	36	32.1		13	20.3	33	38.8	
No Funding	2	5.4	8	7.1		2	3.1	8	9.4	
Public	12	32.4	43	38.4		31	48.4	24	28.2	
Private	0	0.0	3	2.7		0	0.0	3	3.5	
Other	5	13.5	6	5.4		5	7.8	6	7.1	
Some combination of Public, Private, or Other	8	21.6	16	14.3		13	20.3	11	12.9	
**Statement of conflict**	***N* = 37**		***N* = 112**			***N* = 64**		***N* = 85**		
No Statement	6	16.2	46	41.1	*	19	29.7	33	38.8	
Statement, No Conflict Exists	28	75.7	59	52.7		39	60.9	48	56.5	
Statement, Conflict Exists	3	8.1	7	6.2		6	9.4	4	4.7	
**Protocol availability**	***N* = 29**		***N* = 75**			***N* = 47**		***N* = 57**		
Full Protocol	0	0.0	1	1.4		0	0	1	1.8	
No Protocol	29	100.0	74	98.6		47	100.0	56	98.2	
**Data availability**	***N* = 29**		***N* = 75**			***N* = 47**		***N* = 57**		
Some Data Sharing	9	31.0	10	13.3	*	12	25.5	7	12.3	
No Data Sharing	20	69.0	65	86.7		35	74.5	50	87.7	
**Replication**	***N* = 26**		***N* = 71**			***N* = 44**		***N* = 53**		
Novel Findings	14	53.8	42	59.2		27	61.4	29	54.7	
Replication	0	0.0	5	7.0		0	0.0	5	9.4	
Novel Findings and Replication	2	7.7	8	11.3		5	11.4	5	9.4	
No Statement on Novelty or Replication	10	38.5	16	22.5		12	27.3	14	26.4	
**Article citation**	***N* = 26**		***N* = 71**			***N* = 44**		***N* = 53**		
***Replication of Index Study***										
No Citing Article	26	100.0	69	97.2		44	100.0	51	96.2	
At Least One Citing Article	0	0.0	2	2.8		0	0.0	2	3.8	
***Systematic Review/Meta-Analysis***										
No Citing Article	26	100.0	70	98.6		44	100.0	52	98.1	
At Least One Citing Article, No Data Included	0	0.0	1	1.4		0	0.0	1	1.9	
At Least One Citing Article, Data Excluded	0	0.0	0	0.0		0	0.0	0	0.0	
At Least One Citing Article, Data Included	0	0.0	0	0.0		0	0.0	0	0.0	

^a^ Funding, Statement of conflict, Protocol availability, and Data availability determined using the full text of articles. Replication determined using the abstract and/or introduction.

^b^ *P* values based on Fisher’s exact test *<0.05 and **0.05 to 0.005.

**Abbreviations:** PMCID, PubMed Central reference number; PMCOA, PubMed Central Open Access.

### Indicators based on open access data (PubMed level) only, 2000–2017

Among the 590 articles published between 2000 and 2017 in eligible research fields directly related to biomedicine, 520 were non-PMCOA articles. Among the 520 non-PMCOA articles, 81 (15.6% [12.6% to 19.1%]) had a PMCID, thus a PDF is available for each individually. However, full text XML (i.e., Extensible Markup Language) for these articles cannot be downloaded in bulk. Therefore, 439 articles did not have a full text available in PubMed.

Of the 439 eligible articles, 184 listed funding sources at the full text level. Nearly two-thirds (115 of 184, 62.5% [55.0% to 69.4%]) included some funding information under the “Publication type, MeSH terms, Secondary source ID” tab on PubMed (e.g., “Research Support, Non-US Gov’t”). There were 39 (21.2% [15.7% to 28.0%]) additional articles in which PubMed provided at least one specific funding source (i.e., a specific grant number). None of the articles disclosed competing interests under a “Conflict of interest statement” tab on PubMed.

Among the 263 articles in which protocol or data sharing would be relevant (excluding articles without some form of empirical data, model/modeling studies, and case studies or case series), there was one systematic review with a registered protocol on PROSPERO that included a University of York Centre for Reviews and Dissemination (CRD) number at the abstract level.

There were six articles that either referenced their clinical trial’s identifier, included a link to ClinicalTrials.gov, or stated that a Clinical Trials repository link was available on the journal website. Five of these articles also had clinical trial identifiers at the PubMed level. Among the 11 articles that discussed supplementary data, database identifiers, or claimed that data were available upon request, two referenced data identifiers or accession numbers in their abstract (PMID: 22224476 [GenBank links under the “Publication type, MeSH terms, Secondary source ID” tab on PubMed], 27871817 [included links to the SILVA Database under the “LinkOut—more resources” tab on PubMed]).

Of 252 articles with empirical data (excluding case studies and case series, systematic reviews/meta-analyses, and cost effectiveness/decision analysis studies) published between 2000 and 2017, five did not have an abstract on PubMed. Among the eight articles classified as partial or full replication studies based on information provided in the abstract and/or introduction, four had enough information in the abstract alone to establish whether they were replication studies.

Approximately half (55 of 123, 44.7% [35.8% to 53.9%]) of the articles claiming to present some novel findings based on the abstract and/or introduction could be classified as novel according to the abstract only. Of the 10 articles that had statements of both study novelty and some form of replication, only four could be classified based on the abstract only.

### Comparison of indicators from 2000–2014 and 2015–2017

**Fig 1:**
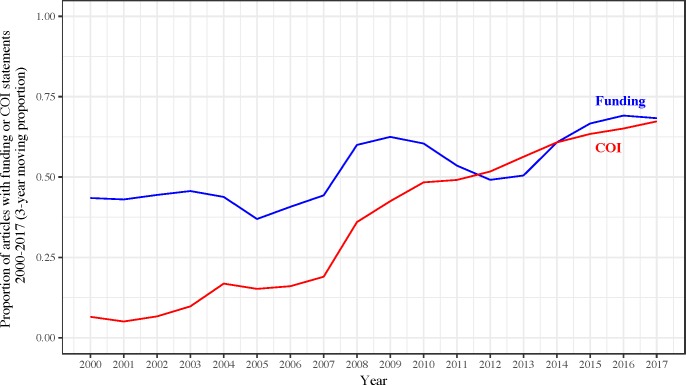
Proportion of articles with funding or COI statements, 2000–2017 (3-year moving proportion). Underlying data for Fig 1 can be found at https://osf.io/3ypdn/. COI, conflicts of interest

**Fig 2:**
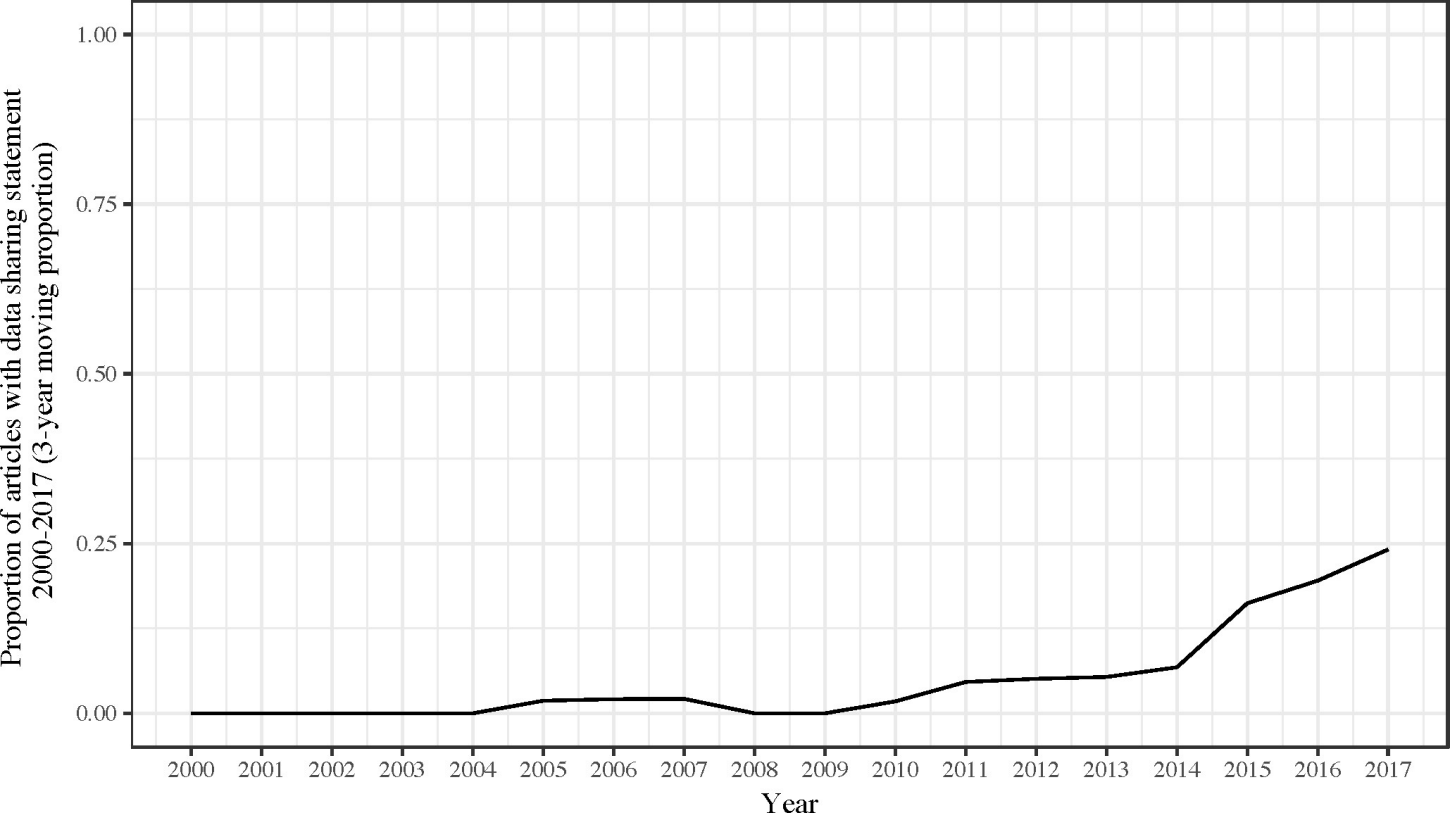
Proportion of articles with data sharing statement, 2000–2017 (3-year moving proportion). Underlying data for Fig 2 can be found at https://osf.io/3ypdn/.

**Fig 3:**
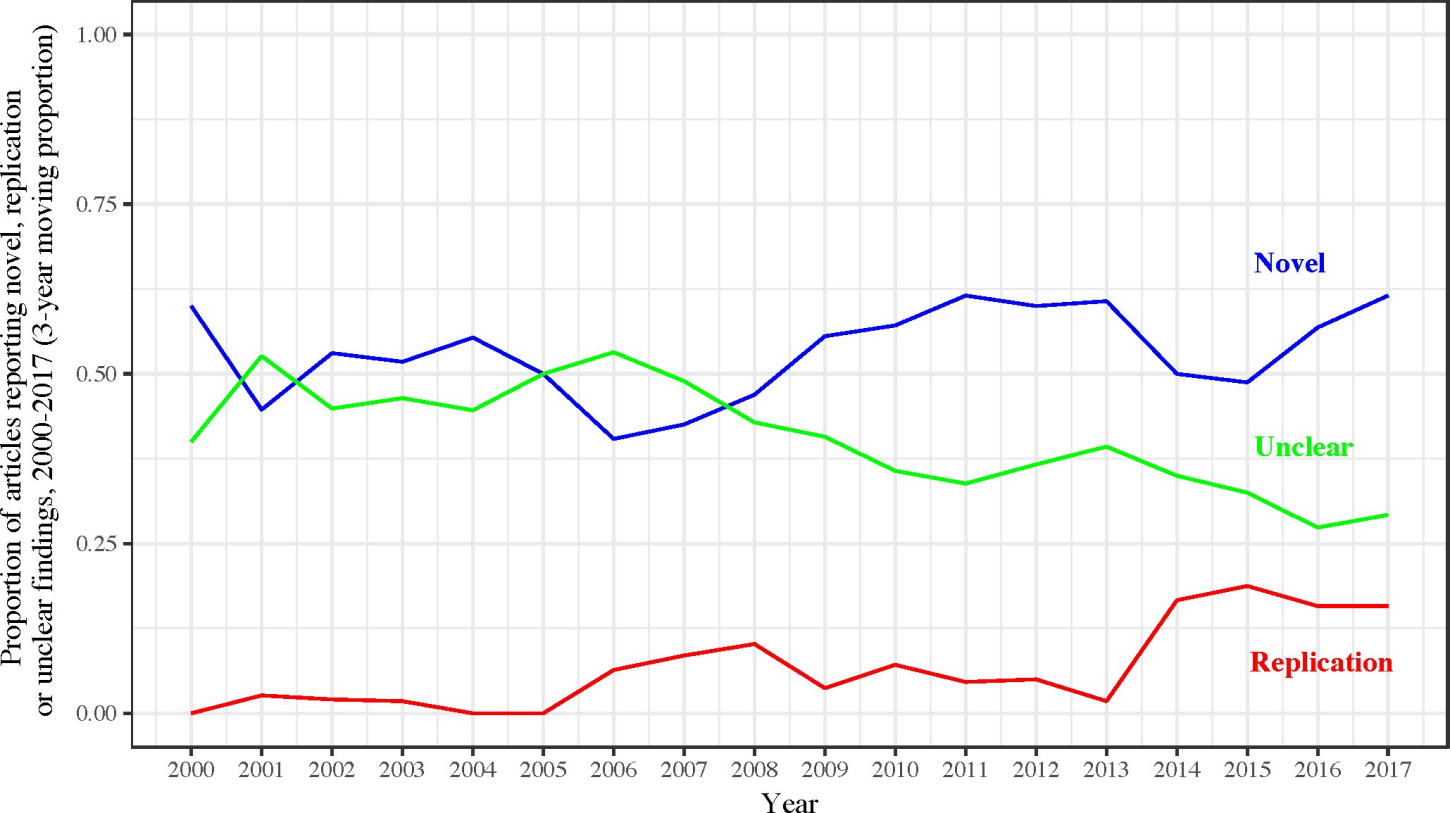
Proportion of articles reporting novel, replication, or unclear findings, 2000–2017 (3-year moving proportion). Underlying data for Fig 3 can be found at https://osf.io/3ypdn/.

A comparison of articles published from 2000–2014 versus 2015–2017 revealed some distinctive patterns (Table 3). Articles published between 2000 and 2014 were less likely to include information related to funding (*p* = 1.4 × 10^−5^). The proportion of articles including information on funding increased over time, with apparently more rapid changes occurring after 2014 (Fig 1). While recently published articles were more likely to contain conflicts of interest statements (*p* = 2.5 × 10^−13^), the proportion of articles with information on conflicts of interests seems to have increased steadily over time (Fig 1). Availability of data substantially increased in 2015–2017 (*p* = 9.7 × 10^−8^), with the proportion of articles including a statement regarding data sharing increasing since 2015 (Fig 2). However, there were no major changes in availability of full protocols. Furthermore, there were more replication attempts published in recent years (either alone or combined with addition novel analyses) (*p* = 3.0 × 10^−4^) (Fig 3). Although the proportion of articles reporting novel findings has remained fairly constant since 2000, there has been a decrease in the proportion of studies with either no or an unclear statement in the abstract and/or introduction about whether there were any novel findings or replication efforts. As expected, fewer articles published in 2015–2017 had already been incorporated in systematic reviews and meta-analyses (given the limited time span available) (*p* = 8.9 × 10^−4^). Open access (PMCOA articles and those with PMCID) proportionally increased substantially in 2015–2017 (*p* = 6.7 × 10^−8^ and *p* = 1.2 × 10^−7^, respectively).

**Table 3:** Comparison of 2000–2014 and 2015–2017 samples.

Variable^a^	Sample				*P* value^b^
	2000–2014		2015–2017		
	*N*	%	*N*	%	
**Funding**	***N* = 441**		***N* = 149**		
No Mention	226	51.3	46	30.9	1.4 × 10^−5^
No Funding	12	2.7	10	6.7	
Public	87	19.7	55	36.9	
Private	19	4.3	3	2.0	
Other	29	6.6	11	7.4	
Some combination of Public, Private, or Other	68	15.4	24	16.1	
**Statement of conflict**	***N* = 441**		***N* = 149**		
No Statement	305	69.2	52	34.9	2.5 × 10^−13^
Statement, No Conflict Exists	110	24.9	87	58.4	
Statement, Conflict Exists	26	5.9	10	6.7	
**Protocol availability**	***N* = 268**		***N* = 104**		
Any Protocol	1	0.4	1	1.0	0.48
No Protocol	267	99.6	103	99.0	
**Data availability**^**c**^	***N* = 268**		***N* = 104**		
Some Data Sharing	5^c^	1.9	19	18.3	9.7 × 10^−8^
No Data Sharing	263	98.1	85	81.7	
**Replication**	***N* = 259**		***N* = 97**		
Novel Findings	133	51.4	56	57.7	3.0 × 10^−4^
Replication	5	1.9	5	5.2	
Novel Findings and Replication	5	1.9	10	10.3	
No Statement on Novelty or Replication	111	42.9	26	26.8	
No Abstract	5	1.9	0	0.0	
**Article citation**	***N* = 259**		***N* = 97**		
***Replication of Index Study***					
No Citing Article	251	96.9	95	97.9	0.73
At Least One Citing Article	8	3.1	2	2.1	
***Systematic Review/Meta-Analysis***					
No Citing Article	221	85.3	96	99.0	8.9 × 10^−4^
At Least One Citing Article, No Data Included	19	7.3	1	1.0	
At Least One Citing Article, Data Excluded	3	1.2	0	0.0	
At Least One Citing Article, Data Included	16	6.2	0	0.0	
**PMCID**	***N* = 441**		***N* = 149**		
Yes	87	19.3	64	42.9	6.7 × 10^−8^
No	354	80.7	85	57.1	
**PMCOA**	***N* = 441**		***N* = 149**		
Yes	33	7.5	37	24.8	1.2 × 10^−7^
No	408	92.5	112	75.2	

^a^Funding, statement of conflict, protocol availability, and data availability determined using the full text of articles. Replication determined using the abstract and/or introduction.

^b^Based on Fisher’s Exact Test.

^c^All were partial data sharing or data sharing statements (complete data set upon request statement, supplementary PDB file provided, demographic and clinical features of patients provided in supplementary table, GenBank links provided, and a link to an original data source used for the study provided).

**Abbreviations:** PMCID, PubMed Central reference number; PMCOA, PubMed Central Open Access.

## Discussion

Our empirical evaluation suggests that there have been improvements over the last few years in certain key indicators of reproducibility and transparency across the full spectrum of published biomedical literature. Approximately two-thirds of articles published in 2015–2017 included information on funding, and a similar proportion included a conflict of interest disclosure statement; these represent major increases versus 2000–2014. Among the biomedical articles in which protocol and data sharing would have been pertinent, nearly one in five articles discussed or publicly shared some portion of their data, while this was hardly ever seen for the sample of articles published between 2000–2014. However, only one article in the 2015–2017 sample referenced a full study protocol. Although the majority of the articles continued to claim some novel findings, approximately 5% were inferred to be replication efforts trying to validate previous knowledge, and 10% included both novel and replication findings, while replication was very rare in 2000–2014. Availability of full texts (either in bulk through PMCOA or more limited in articles with a PMCID but not in the PMCOA) also became more common, although the majority of full texts are still not available. This is important, since open access information reported in the PubMed record without the full text is deficient across several indicators of transparency, such as availability of raw data and conflicts of interest. PubMed records nevertheless included substantial information on funding sources and offered some insights on replication.

We found that approximately one-third of the sample of biomedical articles published between 2015 and 2017 reported no information regarding funding. Although over half of the articles published between 2000 and 2014 did not report any information pertaining to funding [7], our analysis suggests that the proportion of articles with funding statements has increased over time, with possibly more rapid changes since 2014. While the proportion of articles with any information related to potential conflicts of interest disclosures has increased rather steadily over time, likely in response to strengthening of biomedical journal disclosure policies [4, 6, 18], the proportion of articles reporting no conflicts of interest has remained fairly constant and may underestimate the true prevalence of conflicts in biomedical research. Previous estimates suggest that up to 69% of published clinical research articles have some form of financial conflict [18–21]. While disclosure of conflicts of interest has become more common as a result of the uniform forms developed by the ICMJE, which is supported by hundreds of biomedical journals, it is possible that authors are not complying with the guidelines or are unaware of potential conflicts that can impact the design, conduct, and analyses of studies [5].

Approximately 20% of the articles in which data sharing would have been pertinent included a statement related to data sharing. Although one article disclosed that the data were available “on reasonable request,” which could be a result of data sharing statements required by the journal, this does not guarantee that the raw data would be made available to any independent scientist. The majority of the articles with data sharing statements referenced specific data repositories, identifiers, or Excel documents. Although we could verify that the links were functioning, we cannot guarantee the full operational potential and completeness of the data. Moreover, one of the articles was actually a data brief, outlining the raw data for an existing study [22]. Data sharing is a critical component of research transparency and reproducibility, as it allows independent investigators to explore new hypotheses, synthesize evidence across studies, and implement the same experimental methods using the same data (i.e., a replication study) [12]. Our findings indicate that substantial progress been made since our previous evaluation of biomedical articles published between 2000 and 2014, with the proportion of articles with information related to data sharing appearing to have increased since 2014. Although many scientific fields still do not promote open science practices, recent incentives have focused on changing the data sharing culture. For instance, since 2015, author guidelines to promote transparency and reproducibility [6], proposed by Nosek and colleagues, have accumulated hundreds of journal signatories. Moreover, certain journals, such as the PLOS journals, are now requiring “authors to make all data underlying the finding described in their manuscript fully available without restriction, with rare exceptions” [23]. However, journal data sharing policies do not guarantee that investigators will actually share their data [11]. In *PLOS ONE*, in which the policy states that the preferred methods of data sharing is deposition in a repository, only 20% of the data availability statements indicate that data are deposited in a repository [24]. In another empirical evaluation of data from randomized trials published in *PLOS Medicine* and *the BMJ* (both of which require full data availability as a prerequisite to publication), only 46% of the data sets could be retrieved [11]. These findings suggest that more stringent policies or new incentives may be necessary to increase data sharing practices. While some journals have adopted badges to acknowledge open science practices [25], funding agencies can also play a key role in promoting data sharing. For instance, in 2015, the NIH Public Access Plan outlined that the “NIH intends to make public access to digital scientific data the standard for all NIH-funded research” [26].

Although data sharing practices may have shown signs of improvement, only one article in our sample referenced a full study protocol, which does not suggest any material improvement from our previous analysis [7]. The index study was a systematic review with a registered PROSPERO protocol. Systematic review and/or meta-analyses protocol development, registration, and publication has long been promoted [27], but recent evidence suggests that only 5% of systematic reviews of biomedical interventions registered a protocol, and one-third had a protocol publicly available [28]. Prespecified protocols may not be feasible in certain scientific fields or for all types of investigation, in which hypothesis-generated experiments are the norm, and trying to enforce them in such applications would be spurious and could cause investigators to be dishonest about their real intentions. However, when hypotheses and ideas can be prespecified, detailed analytical plans can improve the credibility of research [2, 4, 7, 27, 29]. While protocols may not always be necessary for all articles with detailed methods sections that clearly establish prespecified analyses and outcomes, many journals have word limits that prevent methods sections from including all information necessary for subsequent investigators to repeat a study. Over the last few years, support for preregistration and protocol development has increased and various preregistration and protocol sharing platforms, including the Open Science Framework (http://osf.io) and AsPredicted (http://AsPredicted.org/), have been introduced. On April 4th, 2017, the PLOS journals announced the addition of the protocols.io platform to the guidelines in all of their journals. Protocols.io is an online platform that allows researchers to create and publish protocols. Since 2017, over 200 other journals, including *eLife*, have partnered with protocols.io. These efforts have reduced barriers to sharing and will likely result in improved preregistration and protocol sharing practices among journals in the future.

Similar to our previous evaluation, approximately half of eligible articles clearly claimed to present novel discoveries [7]. Despite claiming novelty on various aspects, it is improbable that the vast majority of articles have truly innovative findings [7, 30]. These results likely reflect the culture of adoring novelty and significance [31, 32]. Investigators are incentivized to say that they do something different and innovative, even when the differences with prior research are subtle or unimportant. However, it is promising that among the sample of eligible articles, five (5%) articles were inferred to be efforts trying to validate some portion of previous knowledge and another 10 included replication components, which is a major increase in the proportion of replication studies identified between 2000 and 2014 [7]. These findings could suggest a gradual shift in the publishing culture toward accepting replications more easily. In particular, some journals have been lowering obstacles for researchers to publish replication studies. For instance, *Research Notes*, a BioMed Central journal, publishes null results and provides “an open access forum for sharing data and useful information” [33]. Similarly, Elsevier has developed a new article type especially for replication studies [34]. Moving forward, the publication of replication studies will be facilitated by emergence of more journals soliciting “non-novel” manuscripts. However, the need to promote the publication of replications does not mean that these should be diverted into separate, distinct journals, which may give the false impression of being second-rate science. In fact, mathematical modeling suggests that replications often are more important than discoveries [35]. We should also acknowledge that an increase in the proportion of articles that use replication-related language may either mean that more replications are performed and/or studies with replication elements are now becoming more readily disclosed as such, while in the past investigators would have tried to sell them as entirely novel.

When we limited our sample to articles without a PMCID and focused on open access data (PubMed level), we found that the vast majority of the eligible articles included some information regarding funding under “Publication type, Mesh terms, Secondary source ID.” However, most of these articles only included nonspecific funding information under the “Publication type, MeSH terms, Secondary source ID” tab on PubMed (e.g., “Research Support, Non-US Gov’t”). In order to establish the potential role that funders could have on the design, analyses, and results from a study, a greater proportion of PubMed abstracts disclosing specific funders and grant numbers will be necessary. Currently, the NLM is transparent about the funding information that is included. In particular, certain grant data “are derived only from the author manuscript submission systems, and not from the published full-text articles” [36, 37].

We identified two articles that had information pertaining to data sharing at the abstract level, and one article that included a protocol registration number at the abstract level. However, none of the eligible articles had a conflict of interest statement tab on PubMed. This is not surprising considering that PubMed only recently announced their new conflicts of interest disclosure policy. Currently, PubMed relies on journal publishers to provide the disclosure information. Moving forward, additional efforts may be necessary to ensure that journals provide all of the necessary disclosure information and that there is a system for compiling disclosure information for articles that were published before 2017. Overall, we are hopeful that PubMed will continue to improve the coverage of conflicts of interest and of funding. This information is essential to have, even if the full text is not available, as readers may still be influenced by reading the abstracts of articles.

Lastly, we found that it was difficult to identify whether non-PMCID studies were replication efforts using only the information available on PubMed. Only half of the articles claiming or inferred to be replication studies could be classified as replication studies based on the abstract alone. This may be a result of the fact that abstracts are often distorted in order to ensure that results are viewed in a more favorable light [38]. However, only half of the articles claiming to present novel results could be classified as “novel” based on the abstract only. These findings emphasize the importance of open access to scholarly work and wider use of the published literature. However, given that a substantial section of PubMed is likely to remain non-open access, it would be useful in the future if PubMed could routinely add information on whether a study includes elements of replication.

It is likely that most scientists are now aware of the need to respond to the calls to improve research transparency and reproducibility. However, it is possible that many are unsure as to what they need to do or change in concrete and practical terms. This confusion exists in spite of the presence of many reviews and commentaries on the problems related to research transparency. We hope that our report can provide a straight-forward “to-do” list about indicators that are worth improving. Continued monitoring of research practices in the form of an observatory may also help inform those who are involved in developing training programs and research support resources.

### Limitations

Our study has certain limitations. Our evaluation relied on published biomedical research information. Therefore, it is possible that additional protocols, raw data, and clarifications on conflicts or funding could be established by contacting authors, journals, or sponsors. Second, our study relied on published records. This means that we based our determination of novelty on the information reported by investigators. For instance, it is possible that authors may have tried to spin their manuscript as being more novel than it really is in order to ensure publication. Although we used our best judgment to classify articles and two authors discussed uncertainties before agreeing upon a final classification, certain decisions were more subjective [7]. Moreover, since only one author conducted the data abstractions, we were unable to calculate any inter-rater reliability metrics. However, the primary abstractor for these data was the coprimary abstractor in a previous study evaluating the same indicators of transparency and reproducibility in articles published in the biomedical literature [7]. Therefore, the primary abstractor of the current evaluation has had extensive experience analyzing these indicators and had an already streamlined process to do so. Nevertheless, when determining study novelty and replication for articles from diverse biomedical fields, difficulty arose assessing whether study results were actually groundbreaking, full or partial replication efforts, or being fully replicated by subsequent studies. In order to account for these limitations, all uncertainties were discussed by two investigators (JDW and JPAI). Third, we did not perform any sample size calculations. Our study evaluated multiple indicators that were all equally important, and they varied substantially in the proportion to which they were satisfied already by the articles in the 2000–2014 sample. The number of annual published biomedical articles increases at approximately 5% per year, and our sample ensured that 2015–2017 would be as well represented as previous years, accounting also for an increase in the volume of published literature over time. Fourth, we acknowledge that the sampling method for the recent set of articles was not identical to the sampling method for the original set of articles. However, when we applied the new enhanced field-classification method based on article-level classification to the original set of 441 articles, we found that 421 were in common between the original and new classifications. With approximately 95% overlap in biomedical definitions between the two samples, we are confident that our population of articles from which the sample was drawn and the sampling methods are comparable. Fifth, our analyses were based on a random sample of 149 biomedical articles published between 2015 and 2017. Therefore, we were unable to account for potential differences in reporting practices across various fields and subdisciplines. Future evaluations should assess these indicators within specific fields. Improvements over time may reflect improvements within specific fields, across many/all fields, and/or an increased representation of the most transparent fields in the more recent literature. Sixth, it is worth noting that we focused on key indicators of reproducibility and transparency that have been proposed as important to monitor. In particular, these indicators were established based on a series of five papers about research published in the *Lancet* [5]. However, these indicators serve as a proxy for transparency and reproducibility and do not capture all potential areas where open science advances may have been made. Finally, an additional limitation is that this study required manual examination of publications and coding of data. We are hopeful that algorithmic means to extract similar information from full text sources (such as PMCOA) can be developed to enable larger scale analyses in the future.

### Conclusion

Our empirical evaluation of biomedical articles published between 2015 and 2017 suggests that progress has been made improving key indicators of reproducibility and transparency. We found that a greater proportion of articles included information on funding, had a conflicts of interest disclosure statement, discussed or publicly shared some portion of their data, and claimed or were inferred to be replication efforts trying to validate previous knowledge. While clinical trial identification numbers and funding details were often provided on PubMed, details related to data sharing and conflicts of interest statements were generally not disclosed. Although numerous efforts to improve reproducibility have already been adopted by researchers, journals, and funders, additional efforts will be necessary to continue to sensitize key stakeholders in the research enterprise of the importance of continuing to improve these indicators over time.

## Materials and methods

We based the design of this study on a previously published manuscript, which includes a study protocol in “Supporting information” [7]. The definitions of captured indicators in the previous evaluation have been carried forward in the current work.

### Sample of assessed articles

We used a sampling process to generate a new random sample of 155 articles published between 2015 and 2017 and indexed in PubMed. We did not perform any sample size calculations since our study evaluated multiple indicators that were all equally important, and they varied substantially in the proportion to which they were satisfied already by the articles in the 2000–2014 sample. Our sample of 155 articles ensured that 2015–2017 would be as well represented as previous years, accounting for the fact that the number of annual published biomedical articles increases approximately 5% per year (Table 4). The sample of 155 articles for the years 2015–2017 was at least 1.5 times that for any other 3-year period from 2000–2014 (Table 4). Articles classified as a “Journal Article” in PubMed were considered and then ordered randomly. Articles in scientific fields not directly related to biomedical research (defined as Biology/Biotechnology, Medicine, Infectious Disease, Health Sciences, and Brain Sciences) [39] were excluded. Even though these fields may sometimes have repercussions for biomedicine, their transparency practices may differ systematically, and separate evaluation efforts would be necessary [7]. All non-English language articles were then excluded and one investigator (JDW) independently characterized the new sample into seven study categories, as previously described (Box 1) [7]. We also considered a previous sample of 441 English language journal articles published between 2000 and 2014 [7] for a comparison against the newer articles and for combined analyses of indicators in terms of open source data. Sampling for the recent set (2015–2017) of papers was done in a manner to produce a set that, given data availability, was as similar as possible to the original set (2000–2014) to enable comparison. Both sets were chosen randomly based on PubMed identification (PMID) numbers. Although both samples were limited to articles considered to be in biomedical fields, in the current analyses, we used an enhanced field classification process based on article-level classification [40], which allowed for a better categorization of both the 155 new articles and the 441 previous articles. Furthermore, the recent sample was limited to “articles” only. While this information was not in place for the original set, only 19 of the 441 articles in the original set were designated as “letters” in PubMed rather than articles [7].

**Box 1. Study categories [7].**

1. No research (items with no data such as editorials, commentaries, news, comments, and nonsystematic expert reviews).
2. Models/modeling, software, script, or methods without empirical data (other than simulations).
3. Case report or series (humans only, with or without review of the literature).
4. Randomized clinical trials (humans only).
5. Systematic reviews and/or meta-analyses (humans only).
6. Cost effectiveness or decision analysis (humans only).
7. Other (empirical data that includes uncontrolled studies [human], controlled nonrandomized studies [human], or basic science studies).

**Table 4:** Number of articles across 3-year periods.

Years	Number of articles
2000–2002	79
2003–2005	89
2006–2008	79
2009–2011	91
2012–2014	103

In order to determine whether there are different reporting practices among free full text articles, we identified the subset of articles made available through PubMed Central (PMC), a digital repository that archives publicly accessible full text biomedical and life science journal articles. Availability of free access in PMC was based on assignment of a PMC identifier (i.e., PMCID versus non-PMCID articles). We also classified articles based on whether there was a publicly available XML version of the full text of the article in the open access subset of PMC (i.e., PMCOA versus non-PMCOA articles). The XML of the full text of roughly 1.7 million PMCOA articles is available in bulk, which allows for algorithmic analyses of the data at scale, as opposed to one at a time. Since 2015, PMCOA comprises roughly half of PMC articles and over 20% of all PubMed articles [41]. We aimed to compare the key indicators of reproducibility across the different article types.

In order to maintain consistency with our previous evaluation [7], we determined the 2013 impact factor of each publication’s journal. The journal name for the eligible article was searched in InCites Journal Citation Reports. No information was recorded for journals without a 2013 impact factor.

### Assessment of indicators of reproducibility and transparency

As previously reported in our survey of biomedical research published between 2000 and 2014 [7], full articles with data and analyses were examined for statements of conflicts of interest, funding disclosures, and publicly available full protocols and data sets. In particular, we reviewed the final versions of the articles available online. For published articles without data and analyses, only statements of conflict and funding were investigated, since protocols, data sets, and reproducibility were not relevant [7]. These indicators, which were assessed in a previous evaluation of articles published in the biomedical literature between 2000 and 2014 [7], have been proposed as being important to monitor in relation to transparency and reproducibility. According to “Increasing value and reducing waste in research design, conduct, and analysis” [5], one of five papers on “Research: Increasing value, reducing waste” published in the *Lancet*, there are several key issues necessary to improve the research process. Under the recommendations section, the authors note that it is necessary to monitor the proportion of research studies “with publicly available (ideally preregistered) protocol and analysis plans, and proportion with raw data…,” “without conflicts of interests, as attested by declaration statements and then checked by reviewers,” and “undergoing rigorous independent replication and reproducibility checks” [5]. As suggested during peer review, we also determined the proportion of articles with (1) statements related to the sharing of script/code by searching the full text of the articles for the words “supporting,” “supplement,” “appendix,” “code,” and “script,” respectively, and (2) any supplemental materials.

The abstracts and introductions were then evaluated for patterns of reproducibility (e.g., whether the study claimed to be a replication effort [Box 2]), as previously described [7]. Web of Knowledge was utilized to determine whether subsequent citing articles had tried to replicate the analyses and whether data were included in systematic reviews and/or meta-analyses.

**Box 2. Assessment of index study novelty [7].**

1. Based on the abstract and/or introduction, the index article claims that it presents some novel findings.
2. Based on the abstract and/or introduction, the index article clearly claims that it is a replication effort trying to validate previous knowledge, or it is inferred that the index article is a replication trying to validate previous knowledge.
3. Based on the abstract and/or introduction, the index article claims to be both novel and to replicate previous findings.
4. No statement or an unclear statement in the abstract and/or introduction about whether the index article presents a novel finding or replication.
5. No distinct abstract and introduction exists.

### Assessment of indicators based on open access data (PubMed level) only

For all articles without a PMCID (i.e., non-PMCID articles) published between 2000 and 2017, we repeated all assessments of indicators of reproducibility and transparency using information reported at the PubMed level. Key indicators of transparency and reproducibility, such as funding and conflicts of interest statements, are often disclosed in the full text of journal articles. However, many research consumers do not have a subscription to all of the journals catalogued in PubMed. Therefore, we evaluated whether PubMed can be used to identify these indicators among the subset of articles without a free full text. Since articles in the PMCOA subset are of particular interest to meta-researchers who want to download information en masse, we repeated our analyses stratified by whether the XML of the full text was publicly available (i.e., PMCOA versus non-PMCOA articles). We examined the title, abstract, “MeSH terms” tab, and the “LinkOut—more resources” tab on PubMed for each article. This captures the metadata-level information that is available in PubMed. We note that article-level metadata can also be downloaded from PubMed in bulk in various formats and that these metadata are amenable to algorithmic mining of information.

### Statistical analysis

Using descriptive statistics, we characterized the indicators of transparency for the period 2015–2017. A priori established Fisher’s exact tests were used to examine differences between the 2000–2014 and 2015–2017 samples, PMCOA and non-PMCOA articles, and PMCID and non-PMCID articles; all statistical tests were two-tailed. As suggested during peer review of our work, we also analyzed potential changes over time for certain indicators of reproducibility and transparency. In particular, we plotted 3-year moving proportions for indicators with an adequate number of events against time. For instance, for the year 2013, we calculated the proportion of articles with a data sharing statement between 2012 and 2014. These analyses can explore more gradual changes that could have occurred. Analyses were performed using R (Version, 3.2.3: The R Project for Statistical Computing). We used the *P* < 0.005 threshold for statistical significance [2, 42], calling results with *P* values 0.05 to 0.005 suggestive.

## Abbreviations

CRD: Centre for Reviews and Dissemination
ICMJE: International Committee of Medical Journal Editors
NIH: National Institutes of Health
NLM: National Library of Medicine
NSF: National Science Foundation
PMC: PubMed Central
PMCID: PubMed Central reference number
PMCOA: PubMed Central Open Access
PMID: PubMed identification
XML: extensible markup language

## References

[1] Baker M. 1,500 scientists lift the lid on reproducibility. Nature. 2016;533(7604):452–4. 10.1038/533452a . 27225100 10.1038/533452a

[2] Munafò MR, Nosek BA, Bishop DVM, Button KS, Chambers CD, Percie du Sert N, et al A manifesto for reproducible science. Nature human behavior. 2017;1(0021). 10.1038/s41562-016-0021 PMC7610724 33954258

[3] Wallach JD, Gonsalves GS, Ross JS. Research, regulatory, and clinical decision-making: the importance of scientific integrity. J Clin Epidemiol. 2017 Epub 2017/10/16. 10.1016/j.jclinepi.2017.08.021 . 29042327 10.1016/j.jclinepi.2017.08.021

[4] Ioannidis JP. How to make more published research true. PLoS Med. 2014;11(10):e1001747 10.1371/journal.pmed.1001747 ; PubMed Central PMCID: PMC4204808. 25334033 10.1371/journal.pmed.1001747 PMC4204808

[5] Ioannidis JP, Greenland S, Hlatky MA, Khoury MJ, Macleod MR, Moher D, et al Increasing value and reducing waste in research design, conduct, and analysis. Lancet. 2014;383(9912):166–75. 10.1016/S0140-6736(13)62227-8 ; PubMed Central PMCID: PMC4697939. 24411645 10.1016/S0140-6736(13)62227-8 PMC4697939

[6] Nosek BA, Alter G, Banks GC, Borsboom D, Bowman SD, Breckler SJ, et al SCIENTIFIC STANDARDS. Promoting an open research culture. Science. 2015;348(6242):1422–5. 10.1126/science.aab2374 ; PubMed Central PMCID: PMC4550299. 26113702 10.1126/science.aab2374 PMC4550299

[7] Iqbal SA, Wallach JD, Khoury MJ, Schully SD, Ioannidis JP. Reproducible Research Practices and Transparency across the Biomedical Literature. PLoS Biol. 2016;14(1):e1002333 10.1371/journal.pbio.1002333 ; PubMed Central PMCID: PMC4699702. 26726926 10.1371/journal.pbio.1002333 PMC4699702

[8] Institute of Medicine (IOM). Sharing Clinical Trial Data: Maximizing Benefits, Minimizing Risks. Washington, DC: The National Academies Press 2015. 25590113

[9] Taichman DB, Backus J, Baethge C, Bauchner H, de Leeuw PW, Drazen JM, et al Sharing Clinical Trial Data—A Proposal from the International Committee of Medical Journal Editors. N Engl J Med. 2016;374(4):384–6. Epub 2016/01/20. 10.1056/NEJMe1515172 . 26786954 10.1056/NEJMe1515172

[10] Taichman DB, Sahni P, Pinborg A, Peiperl L, Laine C, James A, et al Data Sharing Statements for Clinical Trials: A Requirement of the International Committee of Medical Journal Editors. JAMA. 2017;317(24):2491–2492. PubMed Central 10.1001/jama.2017.6514 28586895 10.1001/jama.2017.6514

[11] Naudet F, Sakarovitch C, Janiaud P, Cristea I, Fanelli D, Moher D, et al Data sharing and reanalysis of randomized controlled trials in leading biomedical journals with a full data sharing policy: survey of studies published in. BMJ. 2018;360:k400 Epub 2018/02/13. 10.1136/bmj.k400 ; PubMed Central PMCID: PMC5809812. 29440066 10.1136/bmj.k400 PMC5809812

[12] Goodman SN, Fanelli D, Ioannidis JP. What does research reproducibility mean? Sci Transl Med. 2016;8(341):341ps12 10.1126/scitranslmed.aaf5027 . 27252173 10.1126/scitranslmed.aaf5027

[13] Open Science Collaboration. Estimating the reproducibility of psychological science. Science. 2015;349(6251):aac4716 10.1126/science.aac4716 . 26315443 10.1126/science.aac4716

[14] Nosek BA, Errington TM. Making sense of replications. Elife. 2017;6 Epub 2017/01/19. 10.7554/eLife.23383 ; PubMed Central PMCID: PMC5245957. 28100398 10.7554/eLife.23383 PMC5245957

[15] Ioannidis JPA. The Reproducibility Wars: Successful, Unsuccessful, Uninterpretable, Exact, Conceptual, Triangulated, Contested Replication. Clin Chem. 2017;63(5):943–5. Epub 2017/03/15. 10.1373/clinchem.2017.271965 . 28298413 10.1373/clinchem.2017.271965

[16] McDougall JA, Ferucci ED, Glover J, Fraenkel L. Telerheumatology: A Systematic Review. Arthritis Care Res (Hoboken). 2017;69(10):1546–57. Epub 2017/08/22. 10.1002/acr.23153 ; PubMed Central PMCID: PMC5436947. 27863164 10.1002/acr.23153 PMC5436947

[17] Mortuaire G, Leroy X, Vandenhende-Szymanski C, Chevalier D, Thisse AS. Comparison of endoscopic and external resections for sinonasal instestinal-type adenocarcinoma. Eur Arch Otorhinolaryngol. 2016;273(12):4343–50. Epub 2016/06/30. 10.1007/s00405-016-4181-4 . 27363404 10.1007/s00405-016-4181-4

[18] Riechelmann RP, Wang L, O'Carroll A, Krzyzanowska MK. Disclosure of conflicts of interest by authors of clinical trials and editorials in oncology. J Clin Oncol. 2007;25(29):4642–7. 10.1200/JCO.2007.11.2482 . 17925561 10.1200/JCO.2007.11.2482

[19] Jagsi R, Sheets N, Jankovic A, Motomura AR, Amarnath S, Ubel PA. Frequency, nature, effects, and correlates of conflicts of interest in published clinical cancer research. Cancer. 2009;115(12):2783–91. 10.1002/cncr.24315 . 19434666 10.1002/cncr.24315

[20] Bekelman JE, Li Y, Gross CP. Scope and impact of financial conflicts of interest in biomedical research: a systematic review. JAMA. 2003;289(4):454–65. . 12533125 10.1001/jama.289.4.454

[21] NIH. Conflict of Interest in Medical Research, Education, and Practice. Washington (DC): National Academies Press (US); 2009. 20662118

[22] Soler L, Labas V, Thélie A, Teixeira-Gomes AP, Grasseau I, Bouguereau L, et al Data on endogenous chicken sperm peptides and small proteins obtained through Top-Down High Resolution Mass Spectrometry. Data Brief. 2016;8:1421–5. Epub 2016/08/16. 10.1016/j.dib.2016.07.050 ; PubMed Central PMCID: PMC5007419. 27617276 10.1016/j.dib.2016.07.050 PMC5007419

[23] PLoS ONE. Data Availability. Available from: http://journals.plos.org/plosone/s/data-availability.

[24] Federer LM, Belter CW, Joubert DJ, Livinski A, Lu YL, Snyders LN, et al Data sharing in PLoS ONE: An analysis of Data Availability Statements. PLoS ONE. 2018;13(5):e0194768 10.1371/journal.pone.0194768 . 29719004 10.1371/journal.pone.0194768 PMC5931451

[25] Kidwell MC, Lazarević LB, Baranski E, Hardwicke TE, Piechowski S, Falkenberg LS, et al Badges to Acknowledge Open Practices: A Simple, Low-Cost, Effective Method for Increasing Transparency. PLoS Biol. 2016;14(5):e1002456 10.1371/journal.pbio.1002456 ; PubMed Central PMCID: PMC4865119. 27171007 10.1371/journal.pbio.1002456 PMC4865119

[26] National Institutes of Health. Plan for Increasing Access to Scientific Publications and Digital Scientific Data from NIH Funded Scientific Research. February 2015.

[27] Silagy CA, Middleton P, Hopewell S. Publishing protocols of systematic reviews: comparing what was done to what was planned. JAMA. 2002;287(21):2831–4. . 12038926 10.1001/jama.287.21.2831

[28] Page MJ, Altman DG, Shamseer L, McKenzie JE, Ahmadzai N, Wolfe D, et al Reproducible research practices are underused in systematic reviews of biomedical interventions. J Clin Epidemiol. 2018;94:8–18. Epub 2017/11/04. 10.1016/j.jclinepi.2017.10.017 . 29113936 10.1016/j.jclinepi.2017.10.017

[29] Ioannidis JP. Why most published research findings are false. PLoS Med. 2005;2(8):e124 10.1371/journal.pmed.0020124 ; PubMed Central PMCID: PMC1182327. 16060722 10.1371/journal.pmed.0020124 PMC1182327

[30] Small H, Tseng H, Patek M. Discovering discoveries: Identifying biomedical discoveries using citation contexts. Journal of Informetrics. 2017;11(1).

[31] Chavalarias D, Wallach JD, Li AH, Ioannidis JP. Evolution of Reporting P Values in the Biomedical Literature, 1990–2015. JAMA. 2016;315(11):1141–8. 10.1001/jama.2016.1952 . 26978209 10.1001/jama.2016.1952

[32] Nosek BA, Spies JR, Motyl M. Scientific Utopia: II. Restructuring Incentives and Practices to Promote Truth Over Publishability. Perspect Psychol Sci. 2012;7(6):615–31. 10.1177/1745691612459058 . 26168121 10.1177/1745691612459058 PMC10540222

[33] BMC Research Notes. Aims and scope. https://bmcresnotes.biomedcentral.com/.

[34] de Weerd-Wilson D, Gunn W. How Elsevier is breaking down barriers to reproducibility. 2017.

[35] Ioannidis JP. Why replication has more scientific value than original discovery. Behavioral and Brain Sciences. 2018;41:e137. 10.1017/S0140525X18000729 31064545

[36] U.S. National Library of Medicine. Funding Support (Grant) Information in MEDLINE/PubMed. 2018. Available from: https://www.nlm.nih.gov/bsd/funding_support.html.

[37] U.S. National Library of Medicine. Grant Number Information Found in the GR Field in MEDLINE/PubMed. 2018. Available from: https://www.nlm.nih.gov/bsd/grant_acronym.html.

[38] Chiu K, Grundy Q, Bero L. 'Spin' in published biomedical literature: A methodological systematic review. PLoS Biol. 2017;15(9):e2002173 10.1371/journal.pbio.2002173 ; PubMed Central PMCID: PMC5593172. 28892482 10.1371/journal.pbio.2002173 PMC5593172

[39] Börner K, Klavans R, Patek M, Zoss AM, Biberstine JR, Light RP, et al Design and update of a classification system: the UCSD map of science. PLoS ONE. 2012;7(7):e39464 10.1371/journal.pone.0039464 ; PubMed Central PMCID: PMC3395643. 22808037 10.1371/journal.pone.0039464 PMC3395643

[40] Klavans R, Koyack BW. Research portfolio analysis and topic prominence. Journal of Informetrics. 2017;11(4).

[41] Boyack KW, Van Eck NJ, Colavizza G, Waltman L. Characterizing in-text citations in scientific articles: A large-scale analysis. Journal of Informetrics. 2018;12(1):59–73.

[42] Ioannidis JPA. The Proposal to Lower P Value Thresholds to .005. JAMA. 2018;319(14):1429–30. 10.1001/jama.2018.1536 . 29566133 10.1001/jama.2018.1536

